# Correction: Usability, acceptability, and self-reported impact of an innovative hepatitis C risk reduction intervention for men have sex with men: A mixed methods study

**DOI:** 10.1371/journal.pone.0295455

**Published:** 2023-11-30

**Authors:** Tamara Prinsenberg, Joël Illidge, Paul Zantkuijl, Maarten Bedert, Maria Prins, Marc van de r Valk, Udi Davidovich

In the title of this article, there is a word missing between “men” and “have”. The correct title is: Usability, acceptability, and self-reported impact of an innovative hepatitis C risk reduction intervention for men who have sex with men: A mixed methods study. The correct citation is: Prinsenberg T, Illidge J, Zantkuijl P, Bedert M, Prins M, van der Valk M, et al. (2022) Usability, acceptability, and self-reported impact of an innovative hepatitis C risk reduction intervention for men who have sex with men: A mixed methods study. PloS ONE 17(2): e0263654. https://doi.org/10.1371/journal.pone.0263654
