# Supplementary material for: Usability, acceptability, and self-reported impact of an innovative hepatitis C risk reduction intervention for men have sex with men: A mixed methods study
Source: PLoS One. 2022 Feb 18;17(2):e0263654. doi: 10.1371/journal.pone.0263654 (PMC8856530; doi:10.1371/journal.pone.0263654)
Supplement: S1 Appendix — (PDF) [file pone.0263654.s001.pdf]

S1 Appendix

Questionnaire sent to toolbox recipients in Dutch and English

Nederlandse vragenlijst

Introductie en toestemming

We willen graag dat de Toolbox aansluit bij jouw verwachtingen. Daarom vragen we je om een evaluatievragenlijst in te vullen. We vragen onder andere naar jouw mening over de NoMoreC Toolbox, de instructies en jouw reden(en) voor het bestellen van de Toolbox. Alvast bedankt voor jouw input!

Als eerste vragen we je toestemming om jouw antwoorden op onderstaande vragen te verwerken. Uiteraard worden alle gegevens vertrouwelijk behandeld en niet gekoppeld aan jouw naam of (e-mail)adres. Meer informatie over het onderzoek lees je op NoMoreC.nl

- ☐ Ik ga hier mee akkoord

**Kenmerken v/d deelnemers**	**Vragen en antwoordopties**
Leeftijd	Wat is je leeftij?<br>...... jaar
Land van herkomst	Wat is je geboorteland?<br>Keuzelijst met landen
MSM	Ben je een man en heb je seks met mannen?<br>☐ Ja<br>☐ Nee
Hiv-status	Wat is je hiv-status?<br>☐ Hiv-positief (wel hiv)<br>☐ Hiv-negatief (geen hiv), getest in de afgelopen 6 maanden<br>☐ Weet ik niet, ik ben langer dan 6 maanden geleden voor het laatst op hiv getest<br>☐ Weet ik niet, ik ben nog nooit op hiv getest
PrEP gebruik	Gebruik je PrEP (Pre-Exposure Prophylaxis)?<br>☐ Ja<br>☐ Nee
HCV infectie	Is er ooit hepatitis C bij je vastgesteld?<br>☐ Ja, één keer<br>☐ Ja, meerdere keren<br>☐ Nee, nog nooit
**Zorgen om HCV infectie**	**Vragen en antwoordopties**
Zorgen om HCV infectie	Maak jij je zorgen over het oplopen van hepatitis C?<br>☐ Ja, ik maak me heel erg zorgen<br>☐ Ja ik maak me zorgen<br>☐ Ik maak me een beetje zorgen<br>☐ Nee, ik maak me geen zorgen<br>☐ Nee, ik maak me helemaal geen zorgen
**Thema**	**Vragen en antwoordopties**
Redenen voor het verkrijgen van de Toolbox	Om welke reden(en) wilde je de NoMoreC Toolbox in huis hebben? Kies maximaal 3 reden(en) die voor jou het meest van belang waren.<br>☐ Ik wil de kans verkleinen dat ik hepatitis C oploop<br>☐ Ik wil de kans op overdracht van hepatitis C naar anderen verkleinen<br>☐ Ik was nieuwsgierig naar de inhoud van de NoMoreC Toolbox<br>☐ Om aan iemand anders te geven<br>☐ Om te gebruiken tijdens seksfeestjes<br>☐ De NoMoreC Toolbox werd mij door iemand aanbevolen<br>☐ Anders, namelijk ....
Toolbox gebruik	Heb je producten uit de Toolbox gebruikt?<br>☐ Ja<br>☐ Nee

	Welke producten uit de Toolbox heb je gebruikt of uitgeprobeerd? (Je kunt meerdere antwoorden aanvinken)<br>□ Informatieboekje<br>□ Instructiekaart over desinfectie van handen en onderarmen<br>□ Sterillium® med<br>□ Blue Wonder® Desinfectie Reiniger doekjes<br>□ Spuitfles<br>□ Latex handschoenen<br>□ Nitril handschoenen<br>□ Condooms<br>□ Spuiten voor booty bumpen<br>□ Spuiten en naalden voor slammen<br>□ Stericups<br>□ Naaldencontainer
	Heb je het gebruik van deze producten vooraf besproken met je sekspartner(s), bijvoorbeeld bij het (online) chatten of vlak voordat jullie samen seks hadden?<br>□ Ja<br>□ Nee<br>□ Soms wel, soms niet
	Welk(e) product(en) ben je van plan te (blijven) gebruiken in de toekomst?<br>□ (Je kunt meerdere antwoorden aanvinken)<br>□ Informatieboekje<br>□ Instructiekaart over desinfectie van handen en onderarmen<br>□ Sterillium® med<br>□ Blue Wonder® Desinfectie Reiniger doekjesF<br>□ Spuitfles<br>□ Latex handschoenen<br>□ Nitril handschoenen<br>□ Condooms<br>□ Spuiten voor bootybumpen<br>□ Spuiten en naalden voor slammen<br>□ Stericups<br>□ Naaldencontainer<br>□ Weet ik nog niet<br>□ Geen enkel product, omdat …………………
Bruikbaarheid van de Toolbox	Over het algemeen, hoe duidelijk vond je de instructie in de NoMoreC Toolbox voor het gebruik van de producten?<br>□ Heel duidelijk<br>□ Duidelijk<br>□ Niet duidelijk, niet onduidelijk<br>□ Onduidelijk<br>□ Heel onduidelijk<br>□ Weet ik niet, ik heb de instructies niet gelezen
	Hoe duidelijk vond je de instructie over desinfectie?<br>□ Heel duidelijk<br>□ Duidelijk<br>□ Niet duidelijk, niet onduidelijk<br>□ Onduidelijk<br>□ Heel onduidelijk<br>□ Weet ik niet, ik heb de instructie over desinfectie niet gelezen
	Heb je de NoMoreC Toolbox gebruikt om in gesprek te gaan over hepatitis C met de volgende personen?<br>Vrienden<br>□ Ja<br>□ Nee<br>Sekspartner(s)<br>□ Ja<br>□ Nee
Aanvaardbaarheid van de Toolbox	Wat vind je van de volgende stelling: 'Ik vind het goed dat er een doosje met een naaldencontainer en drugsattributen in de NoMoreC Toolbox zit.'<br>□ Helemaal mee eens<br>□ Mee eens<br>□ Niet mee eens, niet mee oneens

	□ Mee oneens<br>□ Helemaal mee oneens
	Zou je de NoMoreC Toolbox aanraden aan een vriend of fuckbuddy?<br>□ Zeker wel<br>□ Waarschijnlijk wel<br>□ Misschien<br>□ Waarschijnlijk niet<br>□ Zeker niet
Context van gebruik	In welke setting heb je de producten gebruikt of uitgeprobeerd? (Meerdere antwoorden zijn mogelijk)<br>□ Bij één-op-één seks<br>□ Bij groepsseks/seksfeestjes (3-4 mannen)<br>□ Bij groepsseks/seksfeestjes (5 of meer mannen)<br>□ Anders, namelijk …………………
Impact op bewustwording	Wat vind je van de volgende stelling? De NoMoreC Toolbox geeft mij inzicht in wat ik kan doen om de kans op hepatitis C-overdracht te verkleinen.<br>□ Helemaal mee eens<br>□ Mee eens<br>□ Niet mee eens, niet mee oneens<br>□ Mee oneens<br>□ Helemaal mee oneens

Einde. Druk op 'verzend' om je antwoorden in te sturen. Heel erg bedankt voor je medewerking!

English questionnaire

Introduction and consent

It's important that the Toolbox meets your needs. That is why we ask you to complete our feedback questionnaire. In it, we ask what you think of the Toolbox and the instructions that came with it, and your reason(s) for ordering the Toolbox. It will only take a few minutes, and we will be grateful for your help!

First, we ask for your permission to analyse your answers to the questions below. All data is treated with complete confidentiality and information is never linked to your name or (e-mail)address. You can read more about our research at www.NoMoreC.nl.

- □ I agree

**Participant characteristics**	**Question & answer options**
Age	How old are you?<br>…… years
Country of origin	Where were you born?<br>Drop down list with countries
MSM	Are you a man who has sex with men?<br>□ Yes<br>□ No
HIV status	Are you HIV-positive or -negative<br>□ HIV-positive (I have HIV)<br>□ HIV-negative (I do not have HIV); I was tested less than 6 months ago<br>□ I don't know; my last HIV test was more than 6 months ago<br>□ I don't know; I have never been tested for HIV
PrEP use	Do you take PrEP (Pre-Exposure Prophylaxis)?<br>□ Yes<br>□ No
Past HCV infection	Have you ever been diagnosed with hepatitis C?<br>□ Yes, once<br>□ Yes, more than once<br>□ No, never
**HCV infection concerns**	**Question & answer options**
Concerns about HCV infection	Are you worried about getting hepatitis C?<br>□ Yes, I am worried

	☐ Yes, I am somewhat worried<br>☐ No, I am not worried
**Theme**	**Question & answer options**
Motives for obtaining toolbox	Why did you decide to get a NoMoreC toolbox?<br>☐ I want to reduce the risk of getting hepatitis C<br>☐ I want to reduce the risk of transmitting hepatitis C to someone else<br>☐ I was curious to know what was in the NoMoreC toolbox<br>☐ To give to someone else<br>☐ To use during sex parties<br>☐ Someone recommended it to me<br>☐ Other, namely…
Toolbox use	Have you used any of the items in the toolbox?<br>☐ Yes<br>☐ No
	Which items in the toolbox have you used or tried out?<br>☐ Information booklet<br>☐ Instructions for disinfecting hands and forearms<br>☐ Sterillium® med<br>☐ Blue Wonder® cleansing and disinfection wipes<br>☐ Spray bottle<br>☐ Latex gloves<br>☐ Nitrile gloves<br>☐ Condoms<br>☐ Syringes for booty bumping<br>☐ Syringes and needles for slamming<br>☐ Stericup® mixing cups<br>☐ Needle container
	Have you talked to your sex partner(s) before actually having sex about using any of these items, for example while chatting (online or face-to-face) or just before having sex?<br>☐ Yes<br>☐ No<br>☐ Only sometimes
	Which of the items do you plan to use or continue using?<br>☐ Information booklet<br>☐ Instructions for disinfecting hands and forearms<br>☐ Sterillium® med<br>☐ Blue Wonder® cleansing and disinfection wipes<br>☐ Spray bottle<br>☐ Latex gloves<br>☐ Nitrile gloves<br>☐ Condoms<br>☐ Syringes for booty bumping<br>☐ Syringes and needles for slamming<br>☐ Stericup® mixing cups<br>☐ Needle container<br>☐ I don't know yet<br>☐ None of the above, because…………….
Toolbox usability	Generally speaking, how clear were the instructions for the items in the NoMoreC Toolbox?<br>☐ Very clear<br>☐ Fairly clear<br>☐ Neither clear nor unclear<br>☐ Fairly unclear<br>☐ Very unclear<br>☐ I don't know; I haven't read the instructions
	How clear were the instructions for disinfection?<br>☐ Very clear<br>☐ Fairly clear<br>☐ Neither clear nor unclear<br>☐ Fairly unclear<br>☐ Very unclear<br>☐ I don't know; I haven't read the instructions for disinfection

	Have you used the NoMoreC Toolbox to start a conversation about hepatitis C with any of the following people?<br>Friends<br>□ Yes<br>□ No<br>Sex partner(s)<br>□ Yes<br>□ No
Toolbox acceptability	Do you agree or disagree with the following statement: 'I think it's good that a box with a needle container and drug equipment was included in the NoMoreC Toolbox.'<br>□ Strongly agree<br>□ Agree<br>□ Neither agree nor disagree<br>□ Disagree<br>□ Strongly disagree
	Would you recommend the NoMoreC Toolbox to a friend or fuck buddy?<br>□ Definitely<br>□ Probably<br>□ Maybe<br>□ Probably not<br>□ Definitely not
Context of use	In which setting have you used or tried out any of the items?<br>□ During one-on-one sex<br>□ During group sex/sex parties (3-4 men)<br>□ During group sex/sex parties (5 or more men)<br>□ Other, namely ....
Impact on awareness	Do you agree or disagree with the following statement: 'The NoMoreC Toolbox gives me a better understanding of what I can do to reduce the risk of hepatitis C transmission.'<br>□ Strongly agree<br>□ Agree<br>□ Neither agree nor disagree<br>□ Disagree<br>□ Strongly disagree
Toolbox usability	Generally speaking, how clear were the instructions for the items in the NoMoreC Toolbox?<br>□ Very clear<br>□ Fairly clear<br>□ Neither clear nor unclear<br>□ Fairly unclear<br>□ Very unclear<br>□ I don't know; I haven't read the instructions
	How clear were the instructions for disinfection?<br>□ Very clear<br>□ Fairly clear<br>□ Neither clear nor unclear<br>□ Fairly unclear<br>□ Very unclear<br>□ I don't know; I haven't read the instructions for disinfection
	Have you used the NoMoreC Toolbox to start a conversation about hepatitis C with any of the following people?<br>Friends<br>□ Yes<br>□ No<br>Sex partner(s)<br>□ Yes<br>□ No

The end. Pres 'send' to submit your answers. Thank you very much for your cooperation!
