# Supplementary material for: Usability, acceptability, and self-reported impact of an innovative hepatitis C risk reduction intervention for men have sex with men: A mixed methods study
Source: PLoS One. 2022 Feb 18;17(2):e0263654. doi: 10.1371/journal.pone.0263654 (PMC8856530; doi:10.1371/journal.pone.0263654)
Supplement: S2 Appendix — (PDF) [file pone.0263654.s002.pdf]

Interview guide Evaluation NoMoreC Toolbox

1. Research questions and study population 2

2. Algemene structuur interviews - Dutch 2

3. Interview guides - Dutch 4

3 a. Toolbox gebruikers – max. 1 uur 4

3 b. Zorg professionals – max. 30 minuten 6

4. Checklist - Dutch 8

5. General interview structure – English 9

6. Interview guides - English 11

6 a. Toolbox users – max. 1 hour 11

6 b. Health care professionals – max. 30 minutes 13

7. Checklist - English 15

Interviewers:
Maarten Bedert
Tamara Prinsenberg

1. Research questions and study population

Research questions:

The aim of this study is to gather in-depth knowledge about the experiences with the toolbox.

1. What are the reasons for ordering a toolbox, intentions to use the products, the actual use, the user experience, motivations and/or barriers to using the toolbox products for MSM at risk of HCV?
2. What are the reasons, motivations and/or barriers to using and distributing the toolbox for health care professionals?
3. What are the reasons, motivations and/or barriers to distributing the toolbox for fetish shop owners?

In addition we aim to understand the experiences with the NoMoreC HCV testing service:

4. What are the reasons, intentions to use, the actual use and motivations and/or barriers to using the NoMoreC HCV testing service for MSM at risk of HCV?

Study population:

1. MSM who ordered, picked-up or received a NoMoreC toolbox.
2. Health care professionals from HIV treatment centers, STI clinic and GPs in Amsterdam who received NoMoreC toolboxes.

2. Algemene structuur interviews - Dutch

Inleiding

- Persoonlijke introductie en introductie NoMoreC
- Doel interview/studie:
  - Het achterhalen van de redenen, motivatie en barrières voor het gebruik van de NoMoreC Toolbox om de Toolbox te kunnen verbeteren en het gebruik te evalueren.
- Er zijn geen foute antwoorden, het gaat om een gesprek waarbij ervaringen worden uitgewisseld.
- Het is niet noodzakelijk alle vragen te beantwoorden. Gezien het thema van het onderzoek kan het zijn dat in de loop van het interview ook persoonlijke en intieme onderwerpen aan bod komen. Daarbij kan ook naar uw persoonlijke opvatting of ervaring gevraagd worden. Vragen niet beantwoorden heeft geen consequenties voor het vervolg van het gesprek.

- Resultaten van de studie worden gebruikt voor wetenschappelijke doeleinden (mogelijke publicatie, data worden geanonimiseerd)

- Tijdsduur is afhankelijk van wat wordt besproken maar gemiddeld tussen 30 min en 1 uur.

- Het interview wordt opgenomen met tape recorder, bezwaar?

- Aanvullend worden notities gemaakt, ook geanonimiseerd, met code.

- Vragen vanuit de deelnemer? Akkoord starten tape?

Informed consent (op tape)
- Inhoud van het gesprek (Gebruik van en ervaringen met de NoMore C Toolbox)
- Toestemming voor gebruik en opname van het gesprek
- Opt-out: ten alle tijden is het mogelijk om het interview te stoppen
- Studie nummer + datum interview

Structuur diepte interview
1. Introductie
2. Informed consent
3. Demografische gegevens/ achtergrond info/ voorgeschiedenis
4. Toolbox
5. Doornemen toolbox
6. C-test (alleen voor MSM)

Afsluiting gesprek
- Checklist: alle onderwerpen beantwoord, hoofdthema's aan bod gekomen?
- Extra toevoeging vanuit de deelnemer?

Evaluatie met deelnemer
- Evaluatie van het gesprek vanuit het perspectief van de deelnemer
- Nog vragen vanuit de deelnemer?
- Incentive

Korte samenvatting van het gesprek
Maken van korte samenvatting gesprek direct na het interview met daarin beschrijving demografische gegeven, en verloop gesprek. Deze combineren met de transcriptie.

Transcriptie
Transcriptie door transcriptiebureau.

3. Interview guides - Dutch

3 a. Toolbox gebruikers – max. 1 uur

Introductie

Mijn naam is ………………………. ik werk als onderzoeker bij de GGD Amsterdam. Ik voer momenteel onderzoek uit naar de ervaringen omtrent de NoMoreC toolbox. Het doel van het onderzoek is, op basis van diepte-interviews, beter te begrijpen wat mensen van de toolbox vinden en hoe ze deze gebruiken en waarom. Met de resultaten van het onderzoek willen wij de toolbox verbeteren.
Ik wil u hartelijk bedanken dat u aan dit onderzoek wilt mee doen. Voordat we beginnen, wil ik graag nog en aantal zaken met u doornemen.

- Het **interview wordt opgenomen**. Dit is nodig om na het gesprek uw antwoorden goed te kunnen begrijpen en verwerken achteraf. Uw anonimiteit wordt hierin gegarandeerd en alles wat u zegt blijft vertrouwelijk. Uw naam of zaken waaraan men u zou kunnen herkennen zullen niet met derden worden gedeeld.

- Het interview **duurt ongeveer een uur** en heeft als doel een goed beeld te krijgen van uw ervaringen met de NoMoreC toolbox. Indien u iets niet duidelijk is of u liever geen antwoord wil geven op; een vraag, zeg dit dan gerust.

- Verder zal ik tijdens ons gesprek **aantekeningen** maken om voor mezelf wat dingen te kunnen onthouden.

Als u hiermee akkoord gaat, zou ik vanaf nu beginnen met de opnames. Heeft u nog vragen? De opnames kunnen op elk moment gestopt worden en u kunt op elk moment vragen stellen.

Informed consent

U heeft aangegeven bereid te zijn om deel te nemen aan een onderzoek met betrekking tot de NoMoreC toolbox. In dit gesprek behandelen we thema's als risico op hepatitis C, voorkomen van een infectie, het gebruik van de toolbox en jouw mening en ervaring met de toolbox. Als u hiermee instemt, gaan we dit interview opnemen. Data die worden verwerkt worden altijd geanonimiseerd waardoor persoonlijke gegevens niet kunnen worden teruggebracht naar u. Het is te allen tijde mogelijk om het interview te stoppen.
Bent u hiermee akkoord?

Achtergrond informatie / Voorgeschiedenis

Studie nummer…..
Ik wil beginnen met wat algemene vragen:

- Geslacht
- Leeftijd
- Herkomst
- Opleiding
- Werk: wat voor werk doe je ?
- Waar woon je?

Vragen over hepatitis C

- Zou je wat kunnen vertellen over jouw ervaring met Hepatitis C?
- Weet je hoe hepatitis C overgedragen wordt?
- Hoe schat jij je kans in dat je hepatitis C oploopt?
- Maak je je hier zorgen over?
    - Waar komt dit gevoel vandaan?
- Laat je je wel eens testen op hepatitis C?

Chronologie Toolbox

Ontvangst toolbox

- Kan je voor mij beschrijven hoe je bij de Toolbox uitgekomen bent?
- Wat was jouw eerste reactie toen je de Toolbox ontving?
- Wanneer was dit?
- Hoe heb je dit ervaren?

Gebruik toolbox: barriers/ facilitators

- Heb je de Toolbox gebruikt?
    - Niet : Wat heeft je weerhouden om het gebruiken?
    - Wel : In welke setting heb je de toolbox gebruikt? Waar en hoe?
        - Heb je hem daarna nog een gebruikt?
        - Kun je nog andere manieren bedenken waarvoor je de Toolbox zou kunnen gebruiken?
        - Heb je hem op een andere manier gebruikt? Kun je dit beschrijven?

- Ben je door de Toolbox in gesprek gekomen over hepatitis C en het voorkomen daarvan?
- Hoe heeft de Toolbox jou geholpen om over hepatitis C te praten?
- Hoe reageerden anderen?
- Heb je de Toolbox anderen aangeraden?

NoMoreC & C-test

- Behalve de Toolbox, ben je bekend met andere onderdelen van het NoMoreC project?
- Heb je wel eens de risicotest van NoMoreC gedaan? (dit is de online vragenlijst om te bepalen of je risico hebt gelopen)
- Heb je wel eens een C-test besteld van NoMoreC? (om thuis te testen op hepatitis C)
    - Niet:
        - Heb je wel eens overwogen om een test te bestellen?
        - Wat vind je ervan dat deze mogelijkheid tot testen bestaat?
        - In welke context zou je daar mogelijk wel gebruik van maken?
        - Is er iets wat jou weerhoudt om een test via NoMoreC te doen?
    - Wel:
        - Wat was de aanleiding om een test te bestellen?
        - Kun je beschrijven hoe het testen via NoMoreC verliep?
        - Hoe heb je het testen via NoMoreC ervaren?
        - Heb je anderen aangeraden te testen via NoMoreC?
- Wat vind je het NoMoreC project?
- Hoe ben je bekend geraakt met het NoMoreC project?

Toolbox doornemen

Pak de Toolbox erbij en benoem de producten uit de doos.

- Welke spullen gebruik je en welke niet?
- Zijn er producten die je mist?
- Hoe sta je er nu in?
- Zijn er producten die je van plan bent te blijven gebruiken?
- In welke context kun je het wel of niet gebruiken? Momenten of plekken?
- Wat zou je er van vinden als er een HIV- zelftest in de doos zou zitten?

Afsluiting

- Hoe heeft u het interview ervaren?
- Ware er onduidelijkheden?
- Heeft u nog vragen aan mij?
- Zijn er nog zaken die u zou willen veranderen?
- Heeft u nog verbeterpunten voor mij als interviewer?

3 b. Zorg professionals – max. 30 minuten

Introductie

Mijn naam is .... ik werk als onderzoeker bij de GGD Amsterdam. Ik voer momenteel onderzoek uit naar de ervaringen omtrent de NoMoreC toolbox. Het doel van het onderzoek is, op basis van diepte-interviews, beter te begrijpen wat mensen van de toolbox vinden en hoe ze deze gebruiken en waarom. Met de resultaten van het onderzoek willen wij de toolbox verbeteren.
Ik wil u hartelijk bedanken dat u aan dit onderzoek wilt mee doen. Voordat we beginnen, wil ik graag nog en aantal zaken met u doornemen.

- Het **interview wordt opgenomen**. Dit is nodig om na het gesprek uw antwoorden goed te kunnen begrijpen en verwerken achteraf. Uw anonimiteit wordt hierin gegarandeerd en alles wat u zegt blijft vertrouwelijk. Uw naam of zaken waaraan men u zou kunnen herkennen zullen niet met derden worden gedeeld.

- Het interview **duurt ongeveer een half uur** en heeft als doel een goed beeld te krijgen van uw ervaringen met de NoMoreC toolbox. Indien u iets niet duidelijk is of u liever geen antwoord wil geven op; een vraag, zeg dit dan gerust.

- Verder zal ik tijdens ons gesprek **aantekeningen** maken om voor mezelf wat dingen te kunnen onthouden.

Als u hiermee akkoord gaat, zou ik vanaf nu beginnen met de opnames. Heeft u nog vragen? De opnames kunnen op elk moment gestopt worden en u kunt op elk moment vragen stellen.

Informed consent

U heeft aangegeven bereid te zijn om deel te nemen aan een onderzoek met betrekking tot de NoMoreC toolbox. In dit gesprek behandelen we thema's als risico op hepatitis C, voorkomen van een infectie, het gebruik van de toolbox en jouw mening en ervaring met de toolbox. Als u hiermee instemt, gaan we dit interview opnemen. Data die worden verwerkt worden altijd geanonimiseerd waardoor persoonlijke gegevens niet kunnen worden teruggebracht naar u. Het is te allen tijde mogelijk om het interview te stoppen.

Achtergrond informatie

- Geslacht
- Leeftijd
- Functie

Vragen over hepatitis C

- Kun je wat vertellen over jouw ervaring met hepatitis C?
- Hoe vaak zie jij mannen die mogelijk risico lopen op HCV?
- Hoe wordt er binnen jouw afdeling/praktijk over HCV gesproken?

NoMoreC

- Heb je de NoMoreC training gevolgd?
- Heb je de E-learning gedaan? Alle 3 de modules?
- Wat vond je van de training?
- Heb je de NoMoreC website bezocht?

Toolbox

Gebruik toolbox

- Kun je beschrijven hoe je de toolbox gebruikt?
- Wat zijn voor jou de triggers om de Toolbox te gebruiken of uit te delen?
- Wanneer en hoe breng jij de Toolbox ter sprake?
- Helpt de Toolbox jou om een gesprek over hepatitis C te starten?
- Helpt de Toolbox jou om risico reductie te bespreken?
- Hoe reageerden mannen als ze de toolbox ontvangen?

Niet gebruikt : Wat heeft je weerhouden om de Toolbox te gebruiken of uit te delen?

Toolbox doornemen

Pak de Toolbox erbij en bekijk samen de producten uit de doos.

- Mis je iets?
- Wat zou je er van vinden als er een HIV- zelftest in de doos zou zitten?

4. Checklist - Dutch

Introductie

- Introductie onderzoeker
- Doel onderzoek
- Eigen ervating: geen foute antwoordn
- Audio-opname en anonym
- Nemen van notities

Informed consent

Nummer, opname, vrijwillige deelname en mogelijkheid om te stoppen, gebruik van data

Demografische gegevens (m/v, leeftijd, functie)

Toolbox users

Herkomst, opleiding, woonplaats

- Chronolgie Toolbox
  - Ontvangst toolbox (hoe, wanneer, ervaring)
  - Gebruik toolbox (in welke setting, blijvend gebruik, manier van gebruik, reacties anderen)
- NoMoreC & C-test
  - Bekend met NoMoreC
  - C-test (gebruik, aanleiding, ervaring)
- Toolbox doornemen

Professionals

- NoMoreC
  - Trainging gevolgd, e-learning, website bezocht
- Toolbox
  - Gebruik in praktijk, triggers voor gebruik, reacties
  - Barrières tot gebruik
- Toolbox doornemen

Afsluiting gesprek

5. General interview structure – English

Introduction

- Personal introduction and introduction NoMoreC
- Purpose of interview/study:
- Finding out the reasons, motivation and barriers for using the NoMoreC Toolbox in order to improve the Toolbox and evaluate its use.
- There are no wrong answers, it is a conversation in which experiences are exchanged.
- It is not necessary to answer all questions. Given the theme of the research, personal and intimate topics may also be discussed during the interview. You may also be asked about your personal opinion or experience. Not answering questions has no consequences for the continuation of the conversation.
- Results of the study will be used for scientific purposes (possible publication, data will be anonymised)
- Duration depends on what is being discussed but on average between 30 min and 1 hour.
- The interview will be recorded with a tape recorder, objection?
- In addition, notes are made, also anonymized, with code.
- Questions from the participant? Agree start tape?
-

Informed Consent (on tape)

- Content of the conversation (Use of and experiences with the NoMore C Toolbox)
- Consent to use and record the conversation
- Opt-out: it is possible to stop the interview at any time
- Study number + interview date

Structure depth interview

1. Introduction
2. Informed Consent
3. Demographic data/ background info/ history
4. Toolbox
5. Review toolbox
6. C-Test (for MSM only)

Closing conversation
Checklist: all topics answered, main themes addressed?
Extra addition from the participant?

Evaluation with participant

- Evaluation of the conversation from the participant's perspective
- Any questions from the participant?
- Incentive

Short summary of the conversation

Making a short summary of the conversation immediately after the interview, including a description of demographic data and the course of the conversation. Combine these with the transcription.

Transcription

Transcription by transcription agency.

6. Interview guides - English

6 a. Toolbox users – max. 1 hour

Introduction

My name is .... I work as a researcher at the GGD Amsterdam. I am currently conducting research into the experiences with the NoMoreC toolbox. The aim of the study is, based on in-depth interviews, to better understand what people think of the toolbox and how they use it and why. We want to improve the toolbox with the results of the research.

I would like to thank you very much for your willingness to participate in this study. Before we begin, I would like to go over a few things with you.

- The **interview will be recorded**. This is necessary to be able to properly understand your answers after the conversation and to process them afterwards. Your anonymity will be guaranteed and everything you say remains confidential. Your name or anything that could identify you will not be shared with third parties.

- The interview will take about an hour and aims to get a good picture of your experiences with the NoMoreC toolbox. If something is not clear to you or if you prefer not to answer; any question, feel free to say so.

- I will also take notes during our conversation to help myself remember some things.

If you agree, I would like to start recording now. Do you have any questions? The recordings can be stopped at any time, and you can ask questions at any time.

Informed Consent

You have indicated that you are willing to participate in a study regarding the NoMoreC toolbox. In this conversation we discuss topics such as the risk of hepatitis C, prevention of an infection, the use of the toolbox and your opinion and experience with the toolbox. If you agree, we will record this interview. Data that is processed is always anonymized so that personal data cannot be returned to you. It is possible to stop the interview at any time.

Do you agree?

Background information / History

Study number.....

I want to start with some general questions:

- Sex
- Age
- Origin
- Education
- Work: what kind of work do you do?
- Where do you live?

Questions about hepatitis C

- Could you tell us about your experience with Hepatitis C?
- Do you know how hepatitis C is transmitted?
- How do you estimate your chance of contracting hepatitis C?
- Are you concerned about this?
- Where does this feeling come from?
- Do you ever get tested for hepatitis C?

Timeline Toolbox

Receipt toolbox

- Can you describe to me how you arrived at the Toolbox?
- What was your first reaction when you received the Toolbox?
- When was this?
- How did you experience this?

Use toolbox: barriers/facilitators

• Have you used the Toolbox?

o Not used : What stopped you from using it?

o Used: In which setting did you use the toolbox? Where and how?

- Did you use it after that?
- Can you think of any other ways you could use the Toolbox?
- Did you use it in a different way? Can you describe this?

• Did the Toolbox start a conversation about hepatitis C and its prevention?

• How has the Toolbox helped you to talk about hepatitis C?

• How did others react?

• Have you recommended the Toolbox to others?

NoMoereC & C-Test

• Besides the Toolbox, are you familiar with other parts of the NoMoreC project?

• Have you ever done the NoMoreC risk test? (this is the online questionnaire to determine whether you have been at risk)

• Have you ever ordered a C-test from NoMoreC? (to test for hepatitis C at home)

o Did not order test:

- Have you ever considered ordering a test?
- What do you think about this possibility for testing?
- In what context would you possibly use it?
- Is there anything stopping you from doing a test via NoMoreC?

o Did order test:

- What was the reason for ordering a test?
- Can you describe how the testing via NoMoreC went?
- How did you experience testing via NoMoreC?
- Have you recommended others to test via NoMoreC?

• What do you think of the NoMoreC project?

• How did you get to know the NoMoreC project?

Review toolbox products

Open the Toolbox and describe the products from the toolbox.

- Which items do you use and which items not?
- Are there any products you miss?
- How do you look at these products now?
- Are there any products you plan to continue using?
- In what context can you use it? In what context can you not use it? Moments or places?
- How would you like it if there was an HIV self-test in the box?

Ending the interview

- How did you experience the interview?
- Were there any ambiguities?
- Do you have any questions for me?
- Are there still things you would like to change?
- Do you have any points for improvement for me as an interviewer?

6 b. Health care professionals – max. 30 minutes

Introduction

My name is .... I work as a researcher at the GGD Amsterdam. I am currently conducting research into the experiences with the NoMoreC toolbox. The aim of the research is, based on in-depth interviews, to better understand what people think of the toolbox and how they use it and why. We want to improve the toolbox with the results of the research.

I would like to thank you very much for your willingness to participate in this research. Before we begin, I would like to go over a few things with you.

- The interview will be recorded. This is necessary to be able to properly understand your answers after the conversation and to process them afterwards. Your anonymity is hereby guaranteed and everything you say remains confidential. Your name or things that could identify you will not be shared with third parties.

- The interview lasts about half an hour and aims to get a good picture of your experiences with the NoMoreC toolbox. If something is not clear to you or if you prefer not to answer; any question, feel free to say so.

- I will also take notes during our conversation to help myself remember some things.

If you agree, I'd start shooting from now on. Do you have any questions? The recordings can be stopped at any time and you can ask questions at any time.

Informed consent

You have indicated that you are willing to participate in a study regarding the NoMoreC toolbox. In this conversation we discuss topics such as the risk of hepatitis C, prevention of an infection, the use of the toolbox and your opinion and experience with the toolbox. If you agree, we will record this interview. Data that is processed is always anonymized so that personal data cannot be returned to you. It is possible to stop the interview at any time.

Background information

- Sex
- Age
- Profession

Questions about hepatitis C

- Can you tell us about your experience with hepatitis C?
- How often do you see men who may be at risk for HCV?
- How is HCV discussed in your department/practice?

NoMoreC

- Have you followed the NoMoreC training?
- Have you done the E-learning? All 3 modules?
- What did you think of the training?
- Have you visited the NoMoreC website?

Toolbox

Use toolbox

- Can you describe how you use the toolbox?
- What are the triggers for you to use or distribute the Toolbox?
- When and how do you bring up the Toolbox?
- Does the Toolbox help you start a conversation about hepatitis C?
- Does the Toolbox help you to discuss risk reduction?
- How did men react when they received the toolbox?

Not used : What has stopped you from using or distributing the Toolbox?

Review Toolbox products

Open the Toolbox and describe the products.

- Are you missing something?
- What do you think about including an HIV self-test in the box?

7. Checklist - English

Introduction

- Introduction researcher
- Target research
- Own experience: no wrong answers
- Audio recording and anonymity
- Taking notes

informed consent
Number, recording, voluntary participation and possibility to stop, use of data

Demographics (m/f, age, position)

**Toolbox users**<br><br>Origin, education, place of residence<br><br>• **Chronology Toolbox**<br><br>o Receipt toolbox (how, when, experience)<br><br>o Use toolbox (in which setting, continued use, way of use, reactions others)<br><br>• **NoMoreC & C test**<br><br>o Familiar with NoMoreC<br><br>o C-test (use, reasons, experience)<br><br>• **Review toolbox**<br><br>o Go through toolbox
**Professionals**<br><br>• **NoMoreC**<br><br>o Training followed, e-learning, website visited<br><br>• **Toolbox**<br><br>o Use in practice, triggers for use, reactions<br><br>o Barriers to use<br><br>• **Review toolbox**

Ending the interview
