# Supplementary material for: Usability, acceptability, and self-reported impact of an innovative hepatitis C risk reduction intervention for men have sex with men: A mixed methods study
Source: PLoS One. 2022 Feb 18;17(2):e0263654. doi: 10.1371/journal.pone.0263654 (PMC8856530; doi:10.1371/journal.pone.0263654)
Supplement: Attachment — Submitted filename: Response to Reviewers.docx [file pone.0263654.s003.docx]

Dear Dr. Bavinton,

Thank you for reviewing our manuscript entitled ‘A mixed methods evaluation of an innovative hepatitis C risk reduction intervention for men who have sex with men’ (manuscript reference number: PONE-D-21-13933) and for giving us the opportunity to submit a revised version. We are grateful to the two reviewers for their useful and constructive comments. We are also very pleased with the recognition of the reviewers provided for the low-threshold character of the paper that was written to be easily readable by audiences with a variety of backgrounds.

This letter includes the reviewers’ comments in black and our response to each in blue font. The changes we have made in the manuscript in order to address these comments are detailed in our responses. A version of the revision with the changes (track and change mode) and a clean version with all changes accepted have been uploaded.

We hope we adequately addressed the issues raised by the reviewers and we sincerely hope that you are willing to publish our work.

Yours sincerely, and on behalf of all co-authors,

Tamara Prinsenberg, corresponding author

Udi Davidovich, last author

**Response to reviewers’ comments**

Title: A mixed methods evaluation of an innovative hepatitis C risk reduction intervention for men who have sex with men

This document includes the reviewers’ comments in black and our response to each in blue font. The changes we have made in the manuscript in order to address these comments are detailed in our responses

As suggested by reviewer #2 (comment 1) the title is changed to: Usability, acceptability, and self-reported impact of an innovative hepatitis C risk reduction intervention for men who have sex with men: a mix methods study

**Reviewer #1:**

This paper details the mixed-methods evaluation of a toolbox provided to MSM at risk of HCV. The methods, analysis and write-up are straightforward and the output is suitable to a range of audiences within academia, clinical settings and the voluntary sector which is great to see. I have a few comments to further strengthen this publication.

1. The authors state that the contents of the tool-box were collectively decided with community members. It would be really helpful to have a greater understanding of this process and the reasons which underpinned intervention design. Co-creation is an important approach and it would help the reader to understand how this worked and what the implications were.

We have added more information on the co-creation process, under ‘Toolbox development and distribution’ in the methods section. In short, it involved: 1) meetings to assess information needs, 2)discuss and generate the intervention ideas, 3) brainstorm sessions on the content of the toolbox, 4) final decision making on the toolbox content and 5)packing of the toolbox. (clean revised version: lines 95-123)

2. A related point, the authors state the IMB model was used as an explanatory framework in describing how the toolbox was formed. It would be helpful to have a greater elaboration on this. Currently it is only mentioned briefly in the introduction and the discussion, and I think there needs to be greater reflection. For example, was there a theory of change or a logic model produced which used on this model to conceptualise how the toolbox could facilitate behaviour change? If so this should be described and presented, perhaps in supplementary information. I’m also not sure how well the results demonstrate the utility of IMB model in this application, more reflection on this in the discussion is required.

The IMB-model was solely used to guide the development of the intervention, and not for evaluation purposes. We have explained the reason for using the IMB model, as the basis of the intervention in the introduction IMB- based interventions have been shown to be effective in preventing risky sexual behaviours among groups exposed to these interventions (lines 82-84).

In addition, we have elaborated on how the IMB model constructs were integrated in the intervention. We have given the aims for each construct and how the different intervention components contribute to reaching these aims. (clean version lines 104- 109) The intervention components and their focus on information, motivation and behavioural skills have been specified in Table 1.

The reviewer is correct that we did not use the IMB-model for the assessment of the effect of the intervention. We concentrated on users’ experiences with the toolbox and report on these under four main themes: 1) Reasons for obtaining toolbox, 2) Toolbox usability and acceptability, 3) Context of use of the toolbox, and 4) Self-reported impact of the toolbox on HCV awareness and behaviour change.

3. Finally, and this is more of a suggestion, the results from the healthcare providers seem to mirror those of the MSM who used the tool-box quite closely. I think these could be merged easily to create a more coherent narrative around the tool-box and its utility. This would also improve legibility.
We combined the perspectives of the toolbox recipients and health care professionals by integrating the results of the health care professionals into theme 1 (reasons for obtaining the toolbox).

I did not proof-read or look for minor grammatical errors.

The manuscript was proof-read by an English native speaker.

Thank you for the opportunity to review this interesting work.

**Reviewer #2:**

Thank you very much for the opportunity to review this manuscript. This manuscript aimed to assess the use of an innovative hepatitis C risk reduction intervention and the impact on behaviors among men who have sex with men. The topic is of great importance, however, the research and result interpretation part may require further adjustment. The comments are as follows:

1. Title: the authors referred this study to an evaluation of the intervention. The study seemed more like a presentation of the current implementation of the intervention rather than an outcome evaluation, as the latter may require more sophisticated research design to conduct a causal inference. It may be better to adjust the title to better fit the research objectives.
We agree that our study does not measure causal inference but rather the self-reported impact of the intervention by its users. Our study is still an evaluation, but not an impact evaluation that aims to establish causality between the intervention and outcomes. We concentrated mainly on the usability, acceptability and the self-reported impact of the intervention by its user. Therefore, we have changed the title to: *Usability, acceptability, and self-reported impact of an innovative hepatitis C risk reduction intervention for men who have sex with men - a mix methods study*

2. Introduction: the authors regarded toolbox as an innovative intervention. It may be more persuasive to add some background of this intervention that indicates how innovative it is by comparing it with prior interventions.

To the best of our knowledge, there is only one other published HCV risk reduction intervention for MSM that also aims to reduce high risk sexual behavior. This intervention was developed in 2016 as part of the Swiss HCVree trial. The intervention is a counselling intervention for HIV/HCV co-infected MSM, to improve self-regulation of risks associated with high risk sexual behaviours and sexualised drug use. The counseling intervention was provided in combination with DAA treatment. We have taken a different approach by targeting MSM based on their sexual practices and not their HIV/HCV-status.

Furthermore, our intervention is innovative because: 1) there is a lack of interventions that go beyond promoting condom use alone, 2) it contains practical tools for HCV risk reduction, 3) was co-created with the gay community. We have added this to our introduction.

3. Methods: the authors reported that the toolbox had been designed according to the IMB model. “According to the model, HCV prevention information and motivation each have a direct effect on behavior as well as combined effect through behavioral skills (P4 Lines 83-85). Please further elaborate this sentence by specifying how the IMB model informed the intervention design, what was the specific v and what were the combined effect of this toolbox.

We used the IMB model as a theoretical framework to guide the development of the intervention. We did not use the model to assess the effect of the intervention, we were interested in the users’ experiences with the toolbox. In addition, see the response to comment 2 of reviewer 1.

4. Research design: the authors reported that the objective was to assess the use of an innovative hepatitis C risk reduction intervention and the impact on behaviors. I assumed that the authors mainly concerned the consumers in terms of intervention utilization rather than providers. If it is the case, I suggest the authors focusing on reporting findings regarding consumers attitude and behaviors. Some findings from the interviews with providers could be integrated into main themes, for instance, barriers for intervention utilization

We integrated the perspectives of the professionals in theme 1: Reasons for obtaining the toolbox.

5. Methods: the authors conducted online survey and telephone interview, respectively. Were these two samples mutually exclusive to each other? If not, it may not be appropriate to conduct inter-group comparison.

The reviewer is correct, there could possibly be overlap. We are not able to correct for that overlap since the questionnaires were collected anonymously. Therefore, we decided it would be best to remove the comparison.

6. Results: please move the descriptions on research methods (e.g. “the interviews lasted 30 to 60 minutes” P9 Lines 178-179) to the Methods section.

Study period, number of participants and duration of interviews have been moved from the results to the methods section.

We have amended our Acknowledgements section and Funding Statement to:

**Funding statement**

This study was performed within the MC Free consortium. MC Free is funded by grants from Gilead Sciences, AbbVie, Janssen-Cilag, Merck Sharpe & Dohme, and Roche Diagnostics. The funders had no involvement in the study design, writing of the manuscript, and decision to submit the article for publication. TP and MP report speaker fees and grants from Gilead Sciences, Merck Sharpe & Dohme, and AbbVie paid to their institute. MvdV's institute received unrestricted research grants and consultancy fees from AbbVie, Gilead, Johnson & Johnson, Merck Sharpe & Dohme, and ViiV Health Care.

**Acknowledgements**

We would like to thank all participants and professionals, who shared their experiences with the toolbox with us.

**Data availability**

We have uploaded our data to Figshare, they can be access on:

<https://doi.org/10.6084/m9.figshare.16676872.v1>
