# Supplementary material for: Usability, acceptability, and self-reported impact of an innovative hepatitis C risk reduction intervention for men have sex with men: A mixed methods study
Source: PLoS One. 2022 Feb 18;17(2):e0263654. doi: 10.1371/journal.pone.0263654 (PMC8856530; doi:10.1371/journal.pone.0263654)
Supplement: Attachment — Submitted filename: Response te reviewers.docx [file pone.0263654.s004.docx]

Dear Dr. Bavinton,

Thank you for reviewing our revised manuscript entitled ‘Usability, acceptability, and self-reported impact of an innovative hepatitis C risk reduction intervention for men who have sex with men: a mixed methods study’ (manuscript reference number: PONE-D-21-13933R1). We are pleased to hear that we have adequately addressed the reviewers comments and that you have provisionally accepted the manuscript.

We have gladly taken the opportunity to strengthen the manuscript with the constructive suggestions given by one of the reviewers.

This letter includes the comments of reviewer 3 in black and our response to each in blue font. The changes we have made in the manuscript in order to address these comments are detailed in our responses. A version of the revision with the changes (track and change mode) and a clean version with all changes accepted have been uploaded. All references to page numbers and lines in our response refer to the clean manuscript (unless otherwise stated).

We hope we have adequately incorporated the suggestions given by the reviewer and we sincerely hope that you are willing to publish our work.

Yours sincerely, and on behalf of all co-authors,

Tamara Prinsenberg

**6. Review Comments to the Author**

Reviewer #3: The following mixed-methods study provides perceptions of a toolkit to reduce high-risk sexual behaviours and high-risk behaviours for HCV infection among MSM and health professionals. I have provided some additional comments that I hope will be of benefit.

Introduction: “The NoMoreC toolbox contains practical tools, to encourage HCV
risk reduction in different settings” – Please provide some examples for an audience that is not in the HCV field.

Thank you for this comment. The following examples were added to the introduction on page 4, line 86-88: “Examples of these tools are condoms for safer sex, gloves and hand disinfectant for safer fisting, single use drug equipment for safer chemsex and various cleaning and disinfection products to create safer conditions for group sex.”

Methods: Development of the toolbox with a “group of MSM”. Who are the group of MSM? Are they are community group? If yes, perhaps state this upfront to increase clarity.

The group of MSM consisted of members of the community. The group was specifically formed for the NoMoreC project. To clarify this we added the following sentence to the methods on page 5, lines 99-101: This group was formed specifically for the NoMoreC project and was composed of members of the Amsterdam gay community. It included men at risk of HCV, men who had been HCV-infected in the past, and men who had concerns about becoming infected.”

Methods: “practical risk reduction steps” – Perhaps provide some examples of these for an international audience that is not in the field of HCV.

We have added two examples, on page 5-6, lines 117-124: “An example of these instructions is the step-by-step explanation on how to disinfect hand and forearms: 1) wash with soap to remove all lube from your skin and rinse well; 2) dry your skin, 3) make a cup with one hand and fill it with hand disinfectant; 4) disinfect hands and forearms by rubbing hand disinfectant for at least 30 minutes. Another example is how to disinfect sex toys and anal douches which was explained in 4 steps: 1) Clean sex toys and anal douche with dish-washing liquid to remove all lube and rinse well; 2) mix 1 part bleach with 9 parts cold water; 3) submerge for 5 minutes in the bleach solution: 4) rinse thoroughly with water and wipe dry with a clean towel.”

Methods: “Decisions on the addition of other products to add to the toolbox…” What were the products? (refer to the Figure here as this detailed is provided later).

To clarify this we added a reference to Figure 1 on page 6, line 126.

Also, approximately how many boxes were packed?

The following sentence has been added to page 6, line 135 to clarify this: “A total of 700 toolboxes were packed; 450 in 2018 and an additional 250 in 2019.”

Methods: “Study participants from both studies…” Please reference the studies here.

We changed this to: Study participants of both the quantitative and the qualitative studies were included if they were at least 18 years of age, were a man who had sex with men, had received the toolbox and spoke the Dutch or English language. (Page 8, lines 165-167)

Methods: “Possible impact of the toolbox on their behaviour…” Please provide some examples. Are we referring to just sexual behaviours and/or drug use? Or both? What aspects of behaviour at the authors particularly interested in?
We were interested in both sexual and drug use behaviours and to learn what changes participants had made based on the usage of the toolbox and it’s information. This has been clarified in the manuscript on page 9, lines 194-203 and examples of behavioural changes have been given.

“We asked participants to reflect on the possible impact of the toolbox on their HCV awareness and on the participants’ sexual and drug-use behaviours. We were interested to learn if participants had made any behavioural changes in their sex lives to reduce the HCV infection risk, based on the information in the toolbox, and what these changes were. For example, did participants start cleaning and disinfect dildos and the area where sex takes place with the suggested products? Did participants who use drugs, safely administer their drugs by single use drug equipment and not share their equipment? Did men who enjoy fisting start using gloves and clean and disinfect their hands and forearms immediately after fisting? Furthermore, we asked the participants if the information provided by the toolbox assisted in making a personal HCV risk assessment. To thank the participants for their contributions they received a gift voucher of €20.”

Methods: Was there a recruitment strategy for the qualitative interviews with MSM or was it just anyone who received a toolbox could participate. Please explain.

We have now elaborated on the recruitment strategy in the text on page 8-9, lines 181-187: “Men who had ordered the toolbox online were given the option to leave their email address if they were willing to share their opinion about the toolbox in the future. Men who had received the toolbox in person from a health care professional or had picked it up at a fetish shop were asked if they could be contacted in the future, to share their experience with the toolbox. Those who agreed to be contacted left their email. In February 2020, all toolbox recipients who agreed to be contacted were sent an email outlining the research aims and request for participation in the study. 20 men reacted to the email and agreed to participate in the study”

Methods: How many STI clinics were contacted for a qualitative interview? It states that the researchers reached thematic saturation following a discussion with 7 participants? Is it possible that the intention was to reach more representatives from the clinics? Were their attempts to reach clinic based in both urban and rural areas? What was the sampling/recruitment strategy? Were the clinics sent the toolboxes or did they request the toolboxes?

The NoMoreC project was aimed at reducing HCV transmission among MSM in Amsterdam. Therefore, the toolbox was only distributed by health care professionals from Amsterdam locations (1 STI clinic, 6 HIV treatment centres, 4 GP practices and 1 NGO working with PWUDs) This was described in the last paragraph of the section **Toolbox development and distribution** (page 7-8, lines 157-161)**.** As the toolbox was only distributed in Amsterdam, we could only include professionals working in this area (Urban only). We included 2 professionals from the STI clinic, 4 from the HIV treatment centres and 1 from the NGO working for PWUDs. All toolboxes were sent to the clinics at the start of the project and they were provided with new toolbox when they ran out.

Methods: “Discrepancies between initial codes and themes…” Please modify this sentence to increase clarity. Discrepancies between the codes AND themes? Or discrepancies when coding?

We amended to data analysis section on page 10, lines 217-219 to clarify this to: “Following the independent assessments of the transcripts, the researchers met frequently to compare and refine existing codes until they reached consensus on main themes and codes.”

Methods: The quantitative data analysis is clear. Please consider adding a bit more detail regarding the qualitative data analysis (e.g., could explain what inductive thematic analysis is or provide a couple of references for the reader).

We have provided a reference to our thematic analysis approach (ref 19), on page 10, line 214.

Results: “At risk of hepatitis C”. Please modify to HCV to be consistent throughout.

This has been adjusted.

Results: Theme 2 and Theme 3 both mention that the toolbox was not utilised outside of the home due to its size. Please consider placing into one section so as to not be repetitive.

We have removed this topic from Theme 2 (page 18, lines 383-390 of Manuscript with track changes) and placed it under Theme 3 (page 19, 415-422 of Manuscript with track changes, page 19, 403-410 of clean Manuscript)

Discussion: A health professional did not want to discuss the toolbox due to the safe injecting equipment. One of the strategies implemented by the community group was to have the safe injecting equipment in another box within the box. Did the health professional know this? How might the box be marketed in such a way that the ‘baby does not get thrown out with the bath water’? It serves an important purpose but might not be marketed at all if health professionals are uncomfortable with the harm reduction approach to drug use. Perhaps the creation of two boxes? Or MSM could order a personalised toolbox?

This professional was not a health care professional but a professional who works with people who use drugs. I have added this to the results, to clarify. He did not want to hand out the Toolbox with safe drug use equipment as this could serve as a trigger for his clients who want to stop using drugs.

All health professionals were aware of the separate box with drug use equipment because they were given demonstration toolboxes to assist them in the discussions about HCV risk reduction.

Unfortunately for us it is not possible to offer 2 different toolboxes for logistical reasons.

Discussion: Do the study authors intend to distribute the toolbox at saunas and clubs? (as mentioned in the Results, participants felt uncomfortable carrying the box outside of the home).

We do not intend to distribute the toolbox at saunas and clubs. However, as part of our multi-level NoMoreC intervention we have given tailored advice to saunas and clubs about safe needle disposal, disinfectants and making condoms, fisting gloves and single use gloves available to their clients. This has been described in our previous paper on the design of the intervention (ref 14). In the introduction we have mentioned that providing tailored advice to sex on premises venues was part of our strategy.

Discussion: “…sexual behaviour and risk knowledge form structural barriers that impeded the identification of men at risk of HCV.” This sentence is not clear. Please consider modifying.

We have modified this on page 22, lines 499-500 to: “…consultation protocols that do not offer time or space to thoroughly discuss high-risk sexual behavior and risk knowledge hamper the possibility to identify men at risk of HCV.’’

Limitations: “possible behaviour change over a longer period of time”. How long is it expected that such an intervention would have an impact? Are future studies needed in this area? How can MSM be supported to reduce their risk of HCV infection long-term (peer-based organisations? Trusting therapeutic relationships with their providers?). Also, as mentioned by the authors, presumably individuals in this study would be open to changing their at-risk behaviours. What about persons who were not interested in changing their sexual risk behaviours? What might be some strategies to engage this group?

Yes, future studies are recommended in this area. We have added the following to the discussion on page 23, lines 512-516: “Future studies into the psychosocial, cultural, and personal factors that contribute to long term HCV risk reduction behavior are recommended. In addition, support needs for MSM who are willing to change their behavior and for MSM who are resistant to change are likely to be different. Therefore, studying both groups is essential for the development of effective future interventions.”
